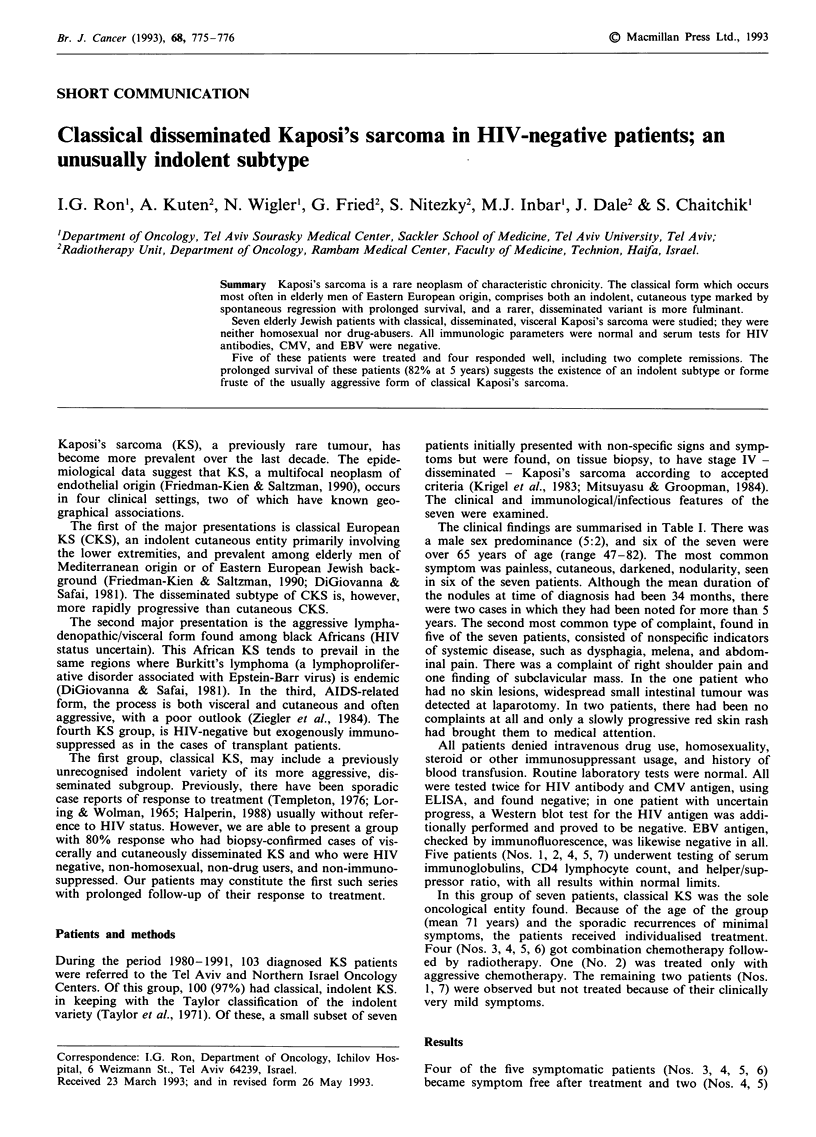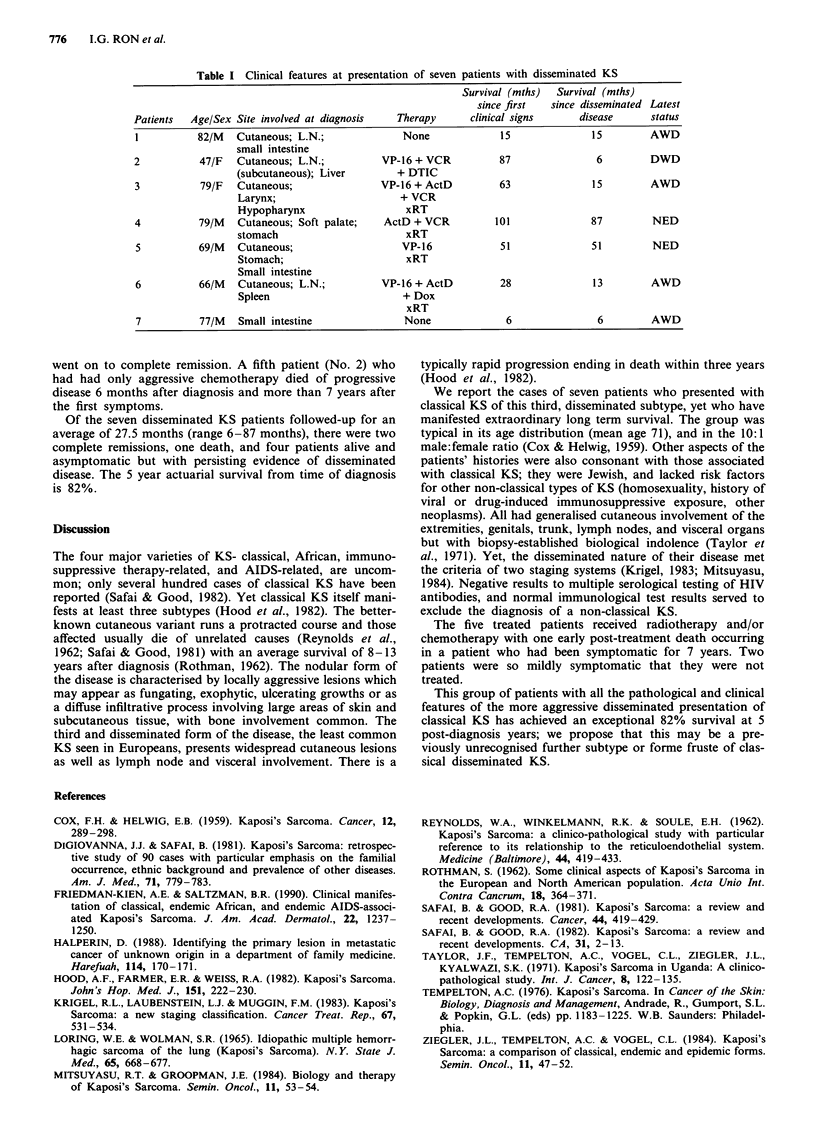# Classical disseminated Kaposi's sarcoma in HIV-negative patients; an unusually indolent subtype.

**DOI:** 10.1038/bjc.1993.426

**Published:** 1993-10

**Authors:** I. G. Ron, A. Kuten, N. Wigler, G. Fried, S. Nitezky, M. J. Inbar, J. Dale, S. Chaitchik

**Affiliations:** Department of Oncology, Tel Aviv Sourasky Medical Center, Sackler School of Medicine, Tel Aviv University, Israel.

## Abstract

Kaposi's sarcoma is a rare neoplasm of characteristic chronicity. The classical form which occurs most often in elderly men of Eastern European origin, comprises both an indolent, cutaneous type marked by spontaneous regression with prolonged survival, and a rarer, disseminated variant is more fulminant. Seven elderly Jewish patients with classical, disseminated, visceral Kaposi's sarcoma were studied; they were neither homosexual nor drug-abusers. All immunologic parameters were normal and serum tests for HIV antibodies, CMV, and EBV were negative. Five of these patients were treated and four responded well, including two complete remissions. The prolonged survival of these patients (82% at 5 years) suggests the existence of an indolent subtype or forme fruste of the usually aggressive form of classical Kaposi's sarcoma.


Br. J. Cancer (1993), 68, 775-776 © Macmillan Press Ltd., 1993

SHORT COMMUNICATION

### Classical disseminated Kaposi's sarcoma in HIV-negative patients; an unusually indolent subtype

I.G. Ron[1], A. Kuten[2], N. Wigler[1], G. Fried[2], S. Nitezky[2], M.J. Inbar[1], J. Dale[2] & S. Chaitchik[1]

[1]*Department of Oncology, Tel Aviv Sourasky Medical Center, Sackler School of Medicine, Tel Aviv University, Tel Aviv;* [2]*Radiotherapy Unit, Department of Oncology, Rambam Medical Center, Faculty of Medicine, Technion, Haifa, Israel.*

**Summary** Kaposi's sarcoma is a rare neoplasm of characteristic chronicity. The classical form which occurs most often in elderly men of Eastern European origin, comprises both an indolent, cutaneous type marked by spontaneous regression with prolonged survival, and a rarer, disseminated variant is more fulminant.

Seven elderly Jewish patients with classical, disseminated, visceral Kaposi's sarcoma were studied; they were neither homosexual nor drug-abusers. All immunologic parameters were normal and serum tests for HIV antibodies, CMV, and EBV were negative.

Five of these patients were treated and four responded well, including two complete remissions. The prolonged survival of these patients (82% at 5 years) suggests the existence of an indolent subtype or forme fruste of the usually aggressive form of classical Kaposi's sarcoma.

Kaposi's sarcoma (KS), a previously rare tumour, has become more prevalent over the last decade. The epidemiological data suggest that KS, a multifocal neoplasm of endothelial origin (Friedman-Kien & Saltzman, 1990), occurs in four clinical settings, two of which have known geographical associations.

The first of the major presentations is classical European KS (CKS), an indolent cutaneous entity primarily involving the lower extremities, and prevalent among elderly men of Mediterranean origin or of Eastern European Jewish background (Friedman-Kien & Saltzman, 1990; DiGiovanna & Safai, 1981). The disseminated subtype of CKS is, however, more rapidly progressive than cutaneous CKS.

The second major presentation is the aggressive lymphadenopathic/visceral form found among black Africans (HIV status uncertain). This African KS tends to prevail in the same regions where Burkitt's lymphoma (a lymphoproliferative disorder associated with Epstein-Barr virus) is endemic (DiGiovanna & Safai, 1981). In the third, AIDS-related form, the process is both visceral and cutaneous and often aggressive, with a poor outlook (Ziegler *et al.*, 1984). The fourth KS group, is HIV-negative but exogenously immunosuppressed as in the cases of transplant patients.

The first group, classical KS, may include a previously unrecognised indolent variety of its more aggressive, disseminated subgroup. Previously, there have been sporadic case reports of response to treatment (Templeton, 1976; Loring & Wolman, 1965; Halperin, 1988) usually without reference to HIV status. However, we are able to present a group with 80% response who had biopsy-confirmed cases of viscerally and cutaneously disseminated KS and who were HIV negative, non-homosexual, non-drug users, and non-immunosuppressed. Our patients may constitute the first such series with prolonged follow-up of their response to treatment.

### Patients and methods

During the period 1980-1991, 103 diagnosed KS patients were referred to the Tel Aviv and Northern Israel Oncology Centers. Of this group, 100 (97%) had classical, indolent KS. in keeping with the Taylor classification of the indolent variety (Taylor *et al.*, 1971). Of these, a small subset of seven patients initially presented with non-specific signs and symptoms but were found, on tissue biopsy, to have stage IV – disseminated – Kaposi's sarcoma according to accepted criteria (Krigel *et al.*, 1983; Mitsuyasu & Groopman, 1984). The clinical and immunological/infectious features of the seven were examined.

The clinical findings are summarised in Table I. There was a male sex predominance (5:2), and six of the seven were over 65 years of age (range 47-82). The most common symptom was painless, cutaneous, darkened, nodularity, seen in six of the seven patients. Although the mean duration of the nodules at time of diagnosis had been 34 months, there were two cases in which they had been noted for more than 5 years. The second most common type of complaint, found in five of the seven patients, consisted of nonspecific indicators of systemic disease, such as dysphagia, melena, and abdominal pain. There was a complaint of right shoulder pain and one finding of subclavicular mass. In the one patient who had no skin lesions, widespread small intestinal tumour was detected at laparotomy. In two patients, there had been no complaints at all and only a slowly progressive red skin rash had brought them to medical attention.

All patients denied intravenous drug use, homosexuality, steroid or other immunosuppressant usage, and history of blood transfusion. Routine laboratory tests were normal. All were tested twice for HIV antibody and CMV antigen, using ELISA, and found negative; in one patient with uncertain progress, a Western blot test for the HIV antigen was additionally performed and proved to be negative. EBV antigen, checked by immunofluorescence, was likewise negative in all. Five patients (Nos. 1, 2, 4, 5, 7) underwent testing of serum immunoglobulins, CD4 lymphocyte count, and helper/suppressor ratio, with all results within normal limits.

In this group of seven patients, classical KS was the sole oncological entity found. Because of the age of the group (mean 71 years) and the sporadic recurrences of minimal symptoms, the patients received individualised treatment. Four (Nos. 3, 4, 5, 6) got combination chemotherapy followed by radiotherapy. One (No. 2) was treated only with aggressive chemotherapy. The remaining two patients (Nos. 1, 7) were observed but not treated because of their clinically very mild symptoms.

### Results

Four of the five symptomatic patients (Nos. 3, 4, 5, 6) became symptom free after treatment and two (Nos. 4, 5)

Correspondence: I.G. Ron, Department of Oncology, Ichilov Hospital, 6 Weizmann St., Tel Aviv 64239, Israel.
Received 23 March 1993; and in revised form 26 May 1993.

Table I Clinical features at presentation of seven patients with disseminated KS

| Patients | Age/Sex | Site involved at diagnosis | Therapy | *Survival (mths) since first clinical signs* | *Survival (mths) since disseminated disease* | *Latest status* |
|---|---|---|---|---|---|---|
| 1 | 82/M | Cutaneous; L.N.; small intestine | None | 15 | 15 | AWD |
| 2 | 47/F | Cutaneous; L.N.; (subcutaneous); Liver | VP-16 + VCR + DTIC | 87 | 6 | DWD |
| 3 | 79/F | Cutaneous; Larynx; Hypopharynx | VP-16 + ActD + VCR xRT | 63 | 15 | AWD |
| 4 | 79/M | Cutaneous; Soft palate; stomach | ActD + VCR xRT | 101 | 87 | NED |
| 5 | 69/M | Cutaneous; Stomach; Small intestine | VP-16 xRT | 51 | 51 | NED |
| 6 | 66/M | Cutaneous; L.N.; Spleen | VP-16 + ActD + Dox xRT | 28 | 13 | AWD |
| 7 | 77/M | Small intestine | None | 6 | 6 | AWD |

went on to complete remission. A fifth patient (No. 2) who had had only aggressive chemotherapy died of progressive disease 6 months after diagnosis and more than 7 years after the first symptoms.

Of the seven disseminated KS patients followed-up for an average of 27.5 months (range 6–87 months), there were two complete remissions, one death, and four patients alive and asymptomatic but with persisting evidence of disseminated disease. The 5 year actuarial survival from time of diagnosis is 82%.

### Discussion

The four major varieties of KS- classical, African, immunosuppressive therapy-related, and AIDS-related, are uncommon; only several hundred cases of classical KS have been reported (Safai & Good, 1982). Yet classical KS itself manifests at least three subtypes (Hood *et al.*, 1982). The better-known cutaneous variant runs a protracted course and those affected usually die of unrelated causes (Reynolds *et al.*, 1962; Safai & Good, 1981) with an average survival of 8–13 years after diagnosis (Rothman, 1962). The nodular form of the disease is characterised by locally aggressive lesions which may appear as fungating, exophytic, ulcerating growths or as a diffuse infiltrative process involving large areas of skin and subcutaneous tissue, with bone involvement common. The third and disseminated form of the disease, the least common KS seen in Europeans, presents widespread cutaneous lesions as well as lymph node and visceral involvement. There is a typically rapid progression ending in death within three years (Hood *et al.*, 1982).

We report the cases of seven patients who presented with classical KS of this third, disseminated subtype, yet who have manifested extraordinary long term survival. The group was typical in its age distribution (mean age 71), and in the 10:1 male:female ratio (Cox & Helwig, 1959). Other aspects of the patients' histories were also consonant with those associated with classical KS; they were Jewish, and lacked risk factors for other non-classical types of KS (homosexuality, history of viral or drug-induced immunosuppressive exposure, other neoplasms). All had generalised cutaneous involvement of the extremities, genitals, trunk, lymph nodes, and visceral organs but with biopsy-established biological indolence (Taylor *et al.*, 1971). Yet, the disseminated nature of their disease met the criteria of two staging systems (Krigel, 1983; Mitsuyasu, 1984). Negative results to multiple serological testing of HIV antibodies, and normal immunological test results served to exclude the diagnosis of a non-classical KS.

The five treated patients received radiotherapy and/or chemotherapy with one early post-treatment death occurring in a patient who had been symptomatic for 7 years. Two patients were so mildly symptomatic that they were not treated.

This group of patients with all the pathological and clinical features of the more aggressive disseminated presentation of classical KS has achieved an exceptional 82% survival at 5 post-diagnosis years; we propose that this may be a previously unrecognised further subtype or forme fruste of classical disseminated KS.

### References

COX, F.H. & HELWIG, E.B. (1959). Kaposi's Sarcoma. *Cancer*, **12**, 289–298.

DIGIOVANNA, J.J. & SAFAI, B. (1981). Kaposi's Sarcoma: retrospective study of 90 cases with particular emphasis on the familial occurrence, ethnic background and prevalence of other diseases. *Am. J. Med.*, **71**, 779–783.

FRIEDMAN-KIEN, A.E. & SALTZMAN, B.R. (1990). Clinical manifestation of classical, endemic African, and endemic AIDS-associated Kaposi's Sarcoma. *J. Am. Acad. Dermatol.*, **22**, 1237–1250.

HALPERIN, D. (1988). Identifying the primary lesion in metastatic cancer of unknown origin in a department of family medicine. *Harefuah*, **114**, 170–171.

HOOD, A.F., FARMER, E.R. & WEISS, R.A. (1982). Kaposi's Sarcoma. *John's Hop. Med. J.*, **151**, 222–230.

KRIGEL, R.L., LAUBENSTEIN, L.J. & MUGGIN, F.M. (1983). Kaposi's Sarcoma: a new staging classification. *Cancer Treat. Rep.*, **67**, 531–534.

LORING, W.E. & WOLMAN, S.R. (1965). Idiopathic multiple hemorrhagic sarcoma of the lung (Kaposi's Sarcoma). *N.Y. State J. Med.*, **65**, 668–677.

MITSUYASU, R.T. & GROOPMAN, J.E. (1984). Biology and therapy of Kaposi's Sarcoma. *Semin. Oncol.*, **11**, 53–54.

REYNOLDS, W.A., WINKELMANN, R.K. & SOULE, E.H. (1962). Kaposi's Sarcoma: a clinico-pathological study with particular reference to its relationship to the reticuloendothelial system. *Medicine (Baltimore)*, **44**, 419–433.

ROTHMAN, S. (1962). Some clinical aspects of Kaposi's Sarcoma in the European and North American population. *Acta Unio Int. Contra Cancrum*, **18**, 364–371.

SAFAI, B. & GOOD, R.A. (1981). Kaposi's Sarcoma: a review and recent developments. *Cancer*, **44**, 419–429.

SAFAI, B. & GOOD, R.A. (1982). Kaposi's Sarcoma: a review and recent developments. *CA*, **31**, 2–13.

TAYLOR, J.F., TEMPELTON, A.C., VOGEL, C.L., ZIEGLER, J.L., KYALWAZI, S.K. (1971). Kaposi's Sarcoma in Uganda: A clinicopathological study. *Int. J. Cancer*, **8**, 122–135.

TEMPELTON, A.C. (1976). Kaposi's Sarcoma. In *Cancer of the Skin: Biology, Diagnosis and Management*, Andrade, R., Gumport, S.L. & Popkin, G.L. (eds) pp. 1183–1225. W.B. Saunders: Philadelphia.

ZIEGLER, J.L., TEMPELTON, A.C. & VOGEL, C.L. (1984). Kaposi's Sarcoma: a comparison of classical, endemic and epidemic forms. *Semin. Oncol.*, **11**, 47–52.